# Gut microbiota shifts favorably with delivery of handwashing with soap and water treatment intervention in a prospective cohort (CHoBI7 trial)

**DOI:** 10.1186/s41043-023-00477-0

**Published:** 2023-12-21

**Authors:** Shirajum Monira, Indrajeet Barman, Fatema Tuz Jubyda, Sk. Imran Ali, Aminul Islam, Kazi Mohammad Zillur Rahman, Mahamud-ur Rashid, Fatema-Tuz Johura, Marzia Sultana, Fatema Zohura, Sazzadul Islam Bhuyian, Tahmina Parvin, David Sack, Tahmeed Ahmed, K M Saif-Ur-Rahman, Maqsud Hossain, Haruo Watanabe, Christine Marie George, Munirul Alam

**Affiliations:** 1 Molecular Ecology and Metagenomics Laboratory, Infectious Diseases Division, icddr,b, (International Centre for Diarrhoeal Diseases Research Bangladesh), Dhaka, Bangladesh; 2 Department of International Health, Johns Hopkins Bloomberg School of Public Health, Baltimore, MD USA; 3 NSU Genome Research Institute, North South University, Dhaka, Bangladesh; 4 National Institutes of Infectious Diseases (NIID), Tokyo, Japan; 5 Evidence Synthesis Ireland and Cochrane Ireland, College of Medicine, Nursing, and Health Sciences, University of Galway, Galway, Ireland

**Keywords:** Cholera, Handwashing with soap, CHoBI7 WASH intervention, Chlorine-treated drinking water, Household-contact children, Gut microbiota

## Abstract

**Background:**

Cholera can result in the expulsion of important microbiota from the gut and result in death if left untreated. The disease transmits mainly via drinking water carrying *Vibrio cholerae*; and household contacts (HHC) of cholera patients are at elevated risk during the first week of infection. The gut microbiota profiles of HHC-children of cholera patients at Dhaka city slums were investigated before (day 0) and after (day 8) delivery of chlorinated water as part of the major study ‘CHoBI7 trial (cholera-hospital-based intervention for 7 days)’.

**Result:**

Results of sequencing and analysis of bacterial community DNA revealed the predominance of two bacterial phyla: Bacteroidetes and Firmicutes at day 0 with a relative abundance of 62 ± 6 (mean ± SEM%) and 32 ± 7, respectively. The pattern reversed at day 8 with a decreased relative abundance of Bacteroidetes (39 ± 12; *p* = 0.034) and an increased abundance of Firmicutes (49 ± 12; *p* = 0.057). Of 65 bacterial families confirmed at day 0, six belonging to Proteobacteria including Vibrionaceae disappeared at day 8. Interestingly, the relative abundance of four Firmicutes families—Lachnospiraceae, Bifidobacteriaceae, Clostridiaceae, and Ruminococcaceae was increased in all five study children at day 8.

**Conclusion:**

The observed exclusion of pathogenic Proteobacteria and enhancement of beneficial Firmicutes in the gut of children delivered with chlorinated water as part of WASH intervention reflect a great promise of the CHoBI7 program in preventing cholera and improving child health.

## Background

The intestinal tract (gut) of humans serves as an important habitat for diverse microbial commensals, also known as microbiota colonizing and adapting to the gut environment creating a state of homeostasis [1, 2]. Microbiota colonization of the gut begins soon after childbirth and continues throughout one’s lifetime depending on the immediate ecosystem they belong to and their overall lifestyle. The gut of a normal individual human can harbor microbiota accounting for more than 10^14^ microbial cells. These commensal microbiota regulate a number of host processes from nutrition and development to immune responses functionally regulating both health and disease [3, 4]. Any qualitative and/ or quantitative change in the gut microbiota composition can alter the set equilibrium resulting in loss of microbiota balance, also known as dysbiosis [5, 6]. With evidence of a causal link between microbiota composition and diverse immuno-compromised infectious and invasive diseases, the microbial repertoire has been a rapidly growing area of research of increasing public health importance in the host-microbe interactions.

Cholera is an acute dehydrating diarrheal disease that can result in death if not treated. *Vibrio cholerae,* the bacteria that causes cholera, is transmitted through contaminated drinking water and poor water, sanitation, and hygiene (WASH) infrastructure and practices in low-resource settings [7]. Cholera remains a major public health problem in many parts of the world and can cause an estimated 4.0 million cases and 95,000 deaths annually [8]. While reported from all around the globe, cholera infections are of primary importance in low-income countries where this disease is endemic due to the lack of safe drinking water and poor WASH infrastructure [9]. Bangladesh continues to have a high disease burden of cholera [8] and related diarrheal diseases. Due to high population density and living in close proximity sharing food and drinking water, the household contacts of cholera patients are at a 100 times higher risk of developing the disease than the general population [10–13].

In Dhaka, Bangladesh millions living in slum areas lack access to improved water sources [14]; and they rely on a communal standpipe which is often an illegal connection to the municipal water supply and highly likely to be fecally contaminated [15, 16]. Globally, unsafe drinking water and poor sanitation and hygiene are among the major contributors to diarrhea, resulting in over 829,000 deaths annually [17], with 19,464 of these deaths occurring in Bangladesh alone [18]. Microbial safe drinking water remains key to save people from diarrhea and related deaths [19]. Frequent use of unsafe water carrying multiple pathogens are responsible for recurrent diarrhea and environmental enteric dysfunction (EED), which is an acquired enteropathy of the small intestine characterized by enteric inflammation, villus blunting and decreased crypt-to-villus ratio that allows pathogenic bacteria to colonize and settle in the gut, as does good bacteria as commensals. EED has been associated with chronic malnutrition (stunting), wasting and reduced vaccine efficacy among children living in low-resource settings [20]. Frequent diarrhea (3–5 episodes/year) and related microbiota dysbiosis [21] are responsible for growth faltering in children who reaches their adulthood as stunted and can have poor cognitive development [22, 23].

The household contacts of cholera patients are at potentially high risk of infection as *V. cholerae* can spread through drinking contaminated water and through person-to-person transmission. We conducted a randomized controlled trial (RCT) in Dhaka city slums of CHoBI7 household contact-children of index cholera patients and delivered a water, sanitation, and hygiene (WASH) intervention including chlorinated water (chlorine tablets) as part of the CHoBI7 to prevent cholera. As in the main RCT, we found drinking chlorine-treated water and handwashing by soapy water during key time points to be highly beneficial for the HHCs as overall *V. cholerae* infection was 47% less in intervention households [24].

Chlorine is a WHO recommended disinfectant and is widely used for water treatment to prevent diarrhea and related infections [25]. In laboratory experiments, we found that the concentration of free chlorine required to inactivate 10^5^ colony-forming units (CFU)/mL of *V. cholerae* serogroups O1 and O139 were estimated to be 0.1 mg/L and 0.2 mg/L, respectively. The concentration of free chlorine generated by a single chlorine tablet (sodium dichloroisocyanurate [33 mg]) after a 30-min reaction time in a 10-L sealed vessel containing Dhaka city municipal supply water was 1.8 mg/L; and the concentration declined to 0.26 mg/L after 24 h [26]. Therefore, it was recommended that CHoBI7 program households use one chlorine tablet every 24 h for household stored drinking water.

In this study, we investigated the gut microbiota composition of the household children of confirmed cholera patients at baseline (Day 0) and one week after (Day 8) initiating delivery of the CHoBI7 program. The aim was to determine if delivery of the CHoBI7 WASH program including chlorine tablets would change microbial communities including cholera bacterium, if any, in the gut.

## Materials and methods

### Study subjects

This study is part of a major study ‘CHoBI7 randomized controlled trial (RCT)’ where handwashing with soap and water treatment intervention were provided to cholera patients and their household contacts in Dhaka city, Bangladesh, from June 2013 to November 2014. The detailed methods for this intervention trial are published elsewhere [24]. As per the study, the intervention arm received chlorine tablets (Aquatabs sodium dichloroisocyanurate, 33 mg; Medentech, Wexford, Ireland, UK), for water treatment, a Topaz container (sealed drinking water vessel with tap) for safe water storage, and a handwashing station. CHoBI7 also included a pictorial module on how cholera can spread through the environment (e.g., contamination of household drinking water sources and stored water), how persons can spread cholera to each other by contaminating food and water in their homes, and instructions on proper handwashing with soap and treatment of water. The control arm received no intervention hardware. Intervention households were instructed to add one chlorine tablet to 10 L water, and store this for up to 24 h.

**Fig. 1:**
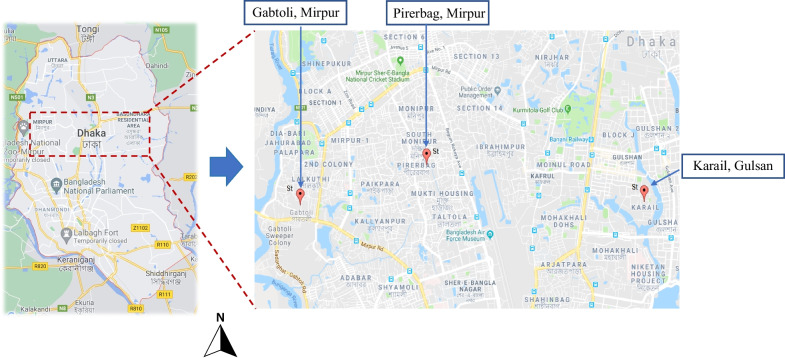
Global positioning system showing distribution of the study households in Dhaka city where cholera patients resided, and their household children were delivered with chlorinated water as part of the cholera-hospital-based water, sanitation, and hygiene (WASH) intervention for 7 days (CHoBI7). Three study children were from Karail slum, one from Gabtoli and the other was from Pirerbag, Mirpur

After one year of the parent study, five children were included in this gut microbiome sub-study residing in the household of culture-confirmed cholera patients admitted in Dhaka icddr,b hospital between May to September 2014. All children resided in slum areas of Dhaka city, Bangladesh. Three children were from Karail slum in Gulshan-1 ward of Dhaka city, one child from Pirerbag, Mirpur, and one from Gabtoli (Fig. 1). The monthly income of parents from moderate socio-economic status ranges from US$ 300 to 500. On the other hand, the parents of the household children from low socio-economic status have a monthly income ranging from US$ 70 to 150. All children in this analysis were 3–7 years of age. After enrolment of cholera patients in CHoBI7 trial those that were already admitted in icddr,b Dhaka hospital, the corresponding household contact of cholera patients were enrolled on the same day or on the following day. Household contacts were defined as those sharing the same cooking pot with the cholera patient for the past three days. Fecal samples were collected from five household children at day 0 and at day 8. After collection of fecal samples, they were immediately preserved at − 20˚C freeze and stored until processing for DNA extraction. We collected a water sample from the household’s stored drinking water at days 0, 1, 5, and 8 to test for the presence of free chlorine, by using a digital colorimeter [26] as per manufacturer’s instruction (Hach, Loveland, CO, USA) which is a proxy measure of water treatment.

### Extraction of total genomic DNA

DNA was extracted from fecal samples according to previously published methods [27]. 125 mg (wet weight) fecal sample was suspended in 625 µl breaking buffer [0.8 mol/L guanidinium isothiocyanate, 4% N-lauroyl sarcosine, 20 mmol/L Tris (pH 8.0), 80 mmol/L sodium phosphate buffer (pH 8.0)] and incubated for 1 h at 70 °C. Afterward, 750 µl glass beads 0.1 mm in diameter (Sigma, St Louis, MO, USA) and 15 mg polyvinylpolypyrrolidone were added. Bacterial cells were lysed in a vortex mixer at high speed (10 cycles consisting of 1-min vortexing and 1-min storage in ice). The mixture was centrifuged at 20,000 g for 3 min at 4 °C. After recovery of the supernatant, the pellet was washed 3 times with 200 µl TENP (50 mm Tris–HCl [pH 8.0], 20 mm EDTA [pH 8.0], 100 mm NaCl, and 1% [w/v] polyvinylpolypyrrolidone). All the four supernatants obtained for a sample were pooled. Nucleic acids were extracted with one volume of phenol. The aqueous phase was washed twice by using chloroform-isoamyl-alcohol (24:1). DNA was precipitated using 100% isopropanol, and the pellet was washed with 70% v/v isopropanol, dried, and resuspended in 50–100 µl of sterile water and stored at − 20 °C. The amount and integrity of DNA were estimated by use of 1% (w/v) agarose gel electrophoresis containing ethidium bromide (1 mg/ml) in 1 X TBE (Tris Borate EDTA).

### Universal primer PCR and illumina MiSeq sequencing

Bacterial 16S ribosomal RNA gene targeted-sequencing was performed. The general bacterial 16S primers used were 341f (CCTACGGGNGGCWGCAG) and 805r (GACTACHVGGGTATCTAATCC), which amplified the V3-V4 region of the 16S rRNA gene. The sequencing library was prepared by following a published protocol [28]. The amplicon libraries were cleaned up with Zymo’s Select-a-Size DNA Clean & Concentrator™

(> 200 fragments were kept), quantified with Tape Station, normalized and pooled together. The final library was quantified with quantitative PCR and sequenced on Illumina MiSeq with v2 reagent kit (500 cycles). The sequencing was performed with > 10% PhiX mix and in paired-end mode.

Raw sequence reads were trimmed with Trimmomatic-0.33 [29]. The two paired end reads in each pair were assembled to construct a complete amplicon sequence with SeqPrep (https://github.com/jstjohn/SeqPrep). Chimeric amplicon sequences were identified and removed with Usearch (v. 6.1) [30] in ref mode against a curated database (http://drive5.com/uchime/rdp_gold.fa). Amplicon sequences smaller than 320 bp were removed. For each sample, 50,000 sequences were randomly sampled to reduce potential bias caused by uneven sampling. These amplicon sequences were compiled, clustered and analyzed with QIIME 1.8.0 [31]. Operational taxonomic units (OTUs) were picked by the workflow of pick_open_reference_otus.py script using GreenGene database (gg_13_8) as the reference database. Relative abundance was measured for each of the bacterium present in the mixed community of each sample. Relative abundance of a particular bacteria is the “percentage of sequences of that bacteria in the mixed bacterial community DNA/sequences”.

### Statistical analysis

Data were analyzed using SPSS version 11.5 (LEAD Technologies Inc., Charlotte, NC, USA). Differences between time points were analyzed for significance using t-tests. Data are presented as mean ± SEM.

## Results

In this study, the gut microbiota of children living in Dhaka city slums was investigated before (day 0) and after (8 days) providing chlorine tablets to make their drinking water germ-free and safe. Residual chlorine in the household drinking water was a measure for chlorination of the drinking water, and handwashing with soap was promoted for seven days as part of a major RCT of cholera hospital-based WASH intervention (CHoBI7). The baseline characteristics of the children are listed in Table 1.

**Table 1:** Baseline characteristics of children included as household contacts of cholera patients

Criteria	Children with cholera (*n* = 5)
Age (yr)	5.1 (3–7)*
Male:Female	1:4
Use of chlorine-treated drinking water during sample collection	Yes
Diarrhea during last 7 days	No
Antibiotic use in last 7 days	No
Socio-economic status	Moderate to low

*Mean (range)

**Fig. 2:**
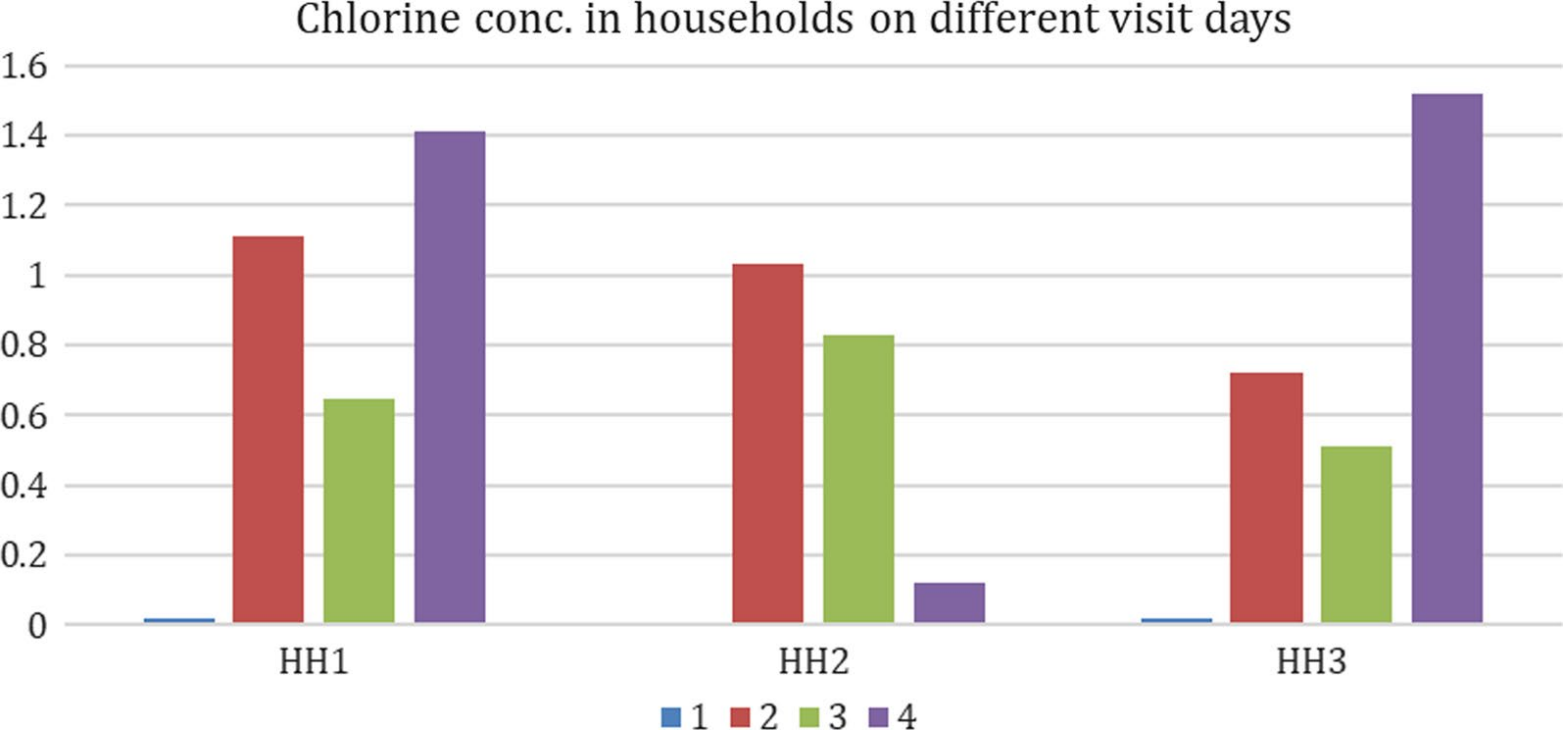
Chlorine concentration found in stored drinking water in the study household before (visit # 1) and after intervention (visit # 2–4). Three study children were enrolled from HH1, and one each from the HH2 and HH3. In HH2, the chlorine concentration was 0 at the visit 1. Water samples from each household were collected from stored drinking water pot during each household visit, and the presence of free chlorine was tested in each water sample using a digital colorimeter [26] following manufacturer’s instruction (Hach, Loveland, CO, USA)

The average free chlorine in stored drinking water over the one-week surveillance period was found to be ≥ 0.66 mg/L in each household (Fig. 2) with the range 0.12–1.52 mg/L. All values were above the cutoff needed to deactivate cholera bacterium *V. cholerae* [26]. None of the study children developed cholera or any other diarrhea infection during this one-week period, and they were in apparently healthy condition.

Illumina MiSeq sequencing of the microbial community DNA in stool samples of the cohort before drinking chlorine-treated water (day 0) found 90–95% of the sequences to be reflecting microbiota. The two most predominant bacterial phyla identified at day 0 include Bacteroidetes and Firmicutes, which showed the relative abundance (mean ± SEM %) of 62 ± 6 and 32 ± 7, respectively. The pattern observed at day 0 changed at day 8 when the relative abundance of bacteria belonging to phylum Bacteroidetes declined (39 ± 12), while those belonging to phylum Firmicutes rose (49 ± 12) and became predominant (*p* = 0.057) in all the study children. The Proteobacteria and Actinobacteria were the next predominant phyla showing the relative abundance of 4 ± 1 and 1 ± 0.7 at day 0; and 4.7 ± 1.9 and 2.3 ± 1.3 at day 8, respectively. The observed decrease in the number of bacteria belonging to the phylum Bacteroidetes after delivering the CHoBI7 WASH program was significant (*p* = 0.034). Bacteria belonging to phyla Euryarchaeota, Verrucomicrobia, Elusimicrobia, Fusobacteria, Spirochaetae, Synergistetes, Tenericutes, and Cyanobacteria did not significantly change their relative abundance as observed at day 0 and day 8.

**Fig. 3:**
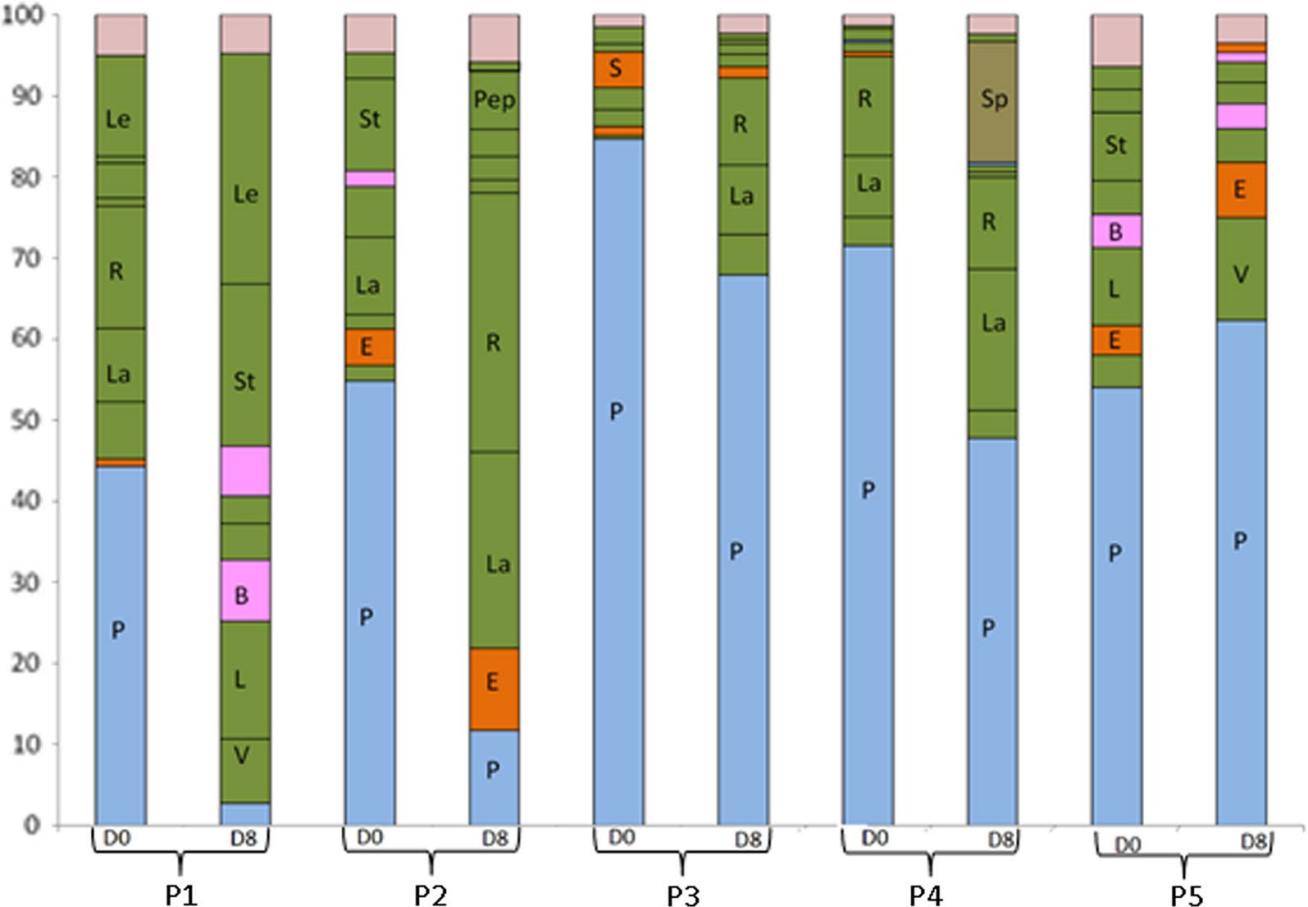
Dominant bacterial groups in the gut of household children at day 0 and day 8 at the taxonomic level of family. Alphabets in the bar reflect bacterial families as indicated below: P, Prevotellaceae; E, Enterobacteriaceae; Le, Leuconostocaceae; R, Ruminococcaceae; La, Lachnospiraceae; St, Streptococcaceae; B, Bifidobacteriaceae; Pep, Peptostreptococcaceae; S, Succinivibrionaceae; Sp, Spirochaetaceae; V, Veillonellaceae; L, Lactobacillaceae

When the top ten bacterial families were considered, substantial inter-individual variation in the dominant bacterial families was observed at day 0 (Fig. 3). The dominant bacteria belonging to the family Prevotellaceae were common for all the five individual children, and bacteria belonging to the family Ruminococcaceae, Streptococcaceae, Succinivibrionaceae, and Lactobacillaceae accounted for the second most predominant bacteria found in the gut of children at day 0.

**Fig. 4:**
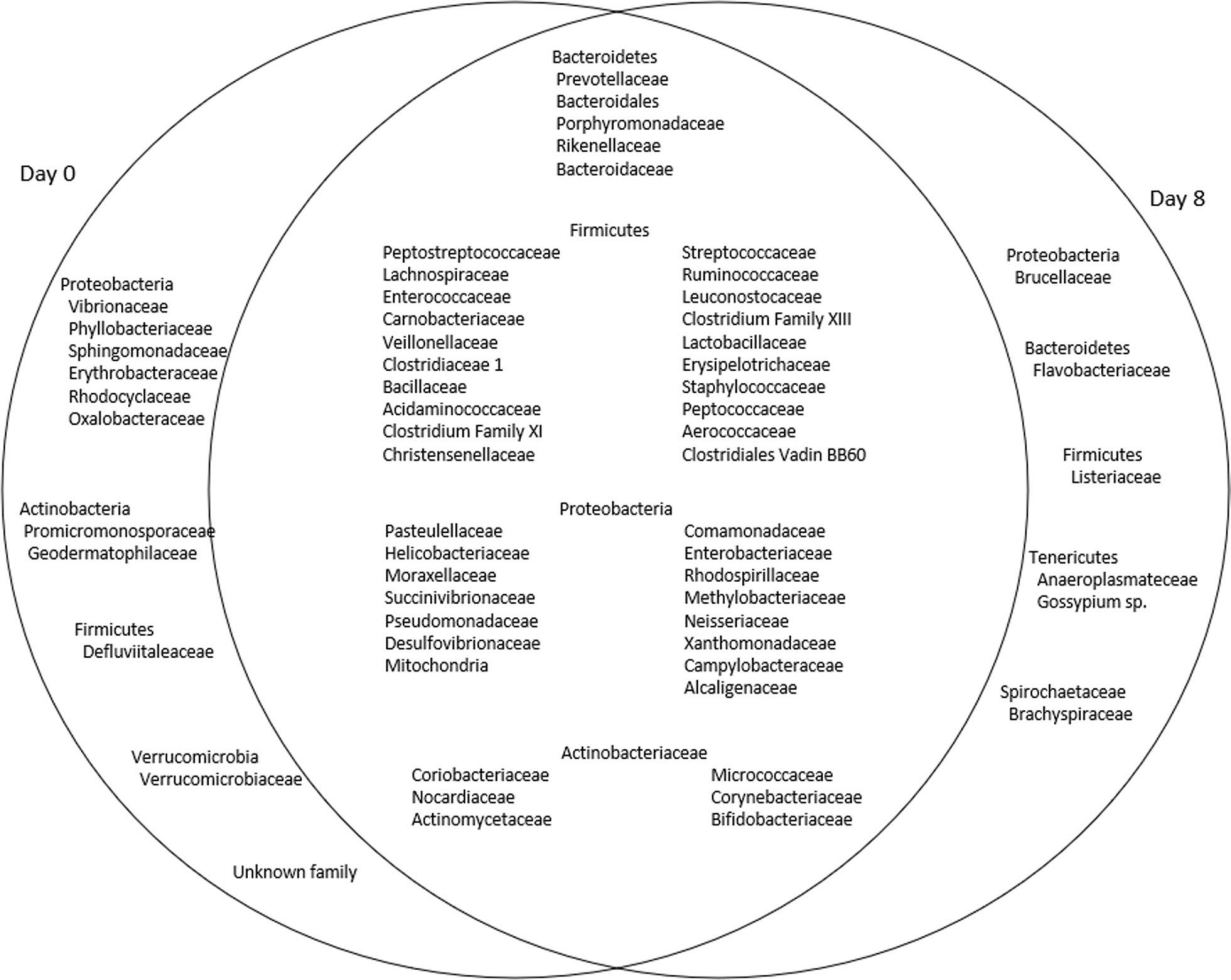
Venn diagram showing the distribution of microbiota belonging to the major phyla at day 0 and at day 8 of the household children of cholera patients. Bacterial families with the relative abundance of ≥ 0.1% were considered for comparison. A total of 65 families were confirmed at day 0 of which 54 were common at both time points, day 0 and day 8, and 6 families were new and acquired as confirmed for household-contact children of cholera patients at day 8, after the delivery of chlorinated water as part of the cholera-hospital-based water, sanitation, and hygiene (WASH) intervention for 7 days (CHoBI7). In the middle circle (overlapped/common) of microbiota, name of 8 genera could not be accommodated, they belonged to the following phyla: Cyanobacteria (2), Euryarchaeota (1), Elusimicrobia (1), Fusobacteria (1), Spirochaetae (1), Synergistetes (1), Tenericutes (1)

Next, we considered families of bacteria belonging to the four major phyla Bacteroidetes, Firmicutes, Proteobacteria, and Actinobacteria, and found significant differences in the gut microbiota profiles for all the five children at day 8. 65 bacterial families were confirmed at day 0. The bacterial families were reduced for all five children at day 8 and only 60 families were present with 6 families that were newly acquired. Total 54 families were common for all five children at both time points (day 0 and day 8) (Fig. 4).

Also, as the cohort of children represented household contacts of hospitalized cholera patients, bacteria belonging to the family *Vibrionaceae* were present in the gut of all five children at day 0, but not after the CHoBI7 WASH program delivery at day 8. Enterobacteriaceae, a family belonging to the phylum Proteobacteria, was common for all five children at the two-time points (day 0 and day 8). The six Proteobacteria families that were found at day 0, but not at day 8 included *Vibrionaceae*, *Phyllobacteriaceae, Sphingomonadaceae, Erythrobacteraceae, Rhodocyclaceae,* and *Oxalobacteraceae*.

Overall results revealed marked differences in the gut microbiota profile in children before and after the CHoBI7 WASH program delivery. When top three bacterial families belonging to the phyla Bacteroidetes, Firmicutes, and Actinobacteria were considered, they showed higher abundance at day 8 compared to that of the phylum Proteobacteria (Table 2).

**Table 2:** Effect of drinking chlorine-treated water on top three bacterial families among children of slums in Dhaka city Bangladesh

Child	Bacterial family					
	Before intervention	Relative abundance (%)		After intervention	Relative abundance (%)	
		Day 0	Day 08		Day 08	Day 0
1	Prevotellaceae	44.3	2.8	Leuconostocaceae	28.4	12.4
	Ruminococcaceae	15.1	3.3	Streptococcaceae	20.0	0.9
	Leuconostocaceae	12.4	28.4	Lactobacillaceae	14.5	7.0
2	Prevotellaceae	54.8	11.8	Ruminococcaceae	32.0	6.3
	Streptococcaceae	11.5	1.6	Lachnospiraceae	24.3	9.5
	Lachnospiraceae	9.5	24.3	Prevotellaceae	11.8	54.8
	Prevotellaceae	84.7	67.9	Prevotellaceae	67.9	84.7
3	Succinivibrionaceae	4.4	1.4	Ruminococcaceae	10.8	2.8
	Ruminococcaceae	2.8	10.8	Lachnospiraceae	8.5	2.0
	Prevotellaceae	71.5	47.8	Prevotellaceae	47.8	71.5
4	Ruminococcaceae	12.3	11.3	Lachnospiraceae	17.4	7.5
	Lachnospiraceae	7.5	17.4	Spirochaetaceae	14.9	XX
	Prevotellaceae	54.1	62.3	Prevotellaceae	62.3	54.1
5	Lactobacillaceae	9.7	4.2	Veillonellaceae	12.7	4.0
	Streptococcaceae	8.4	0.9	Enterobacteriaceae	6.8	3.6

*XX* Not present

**Fig. 5:**
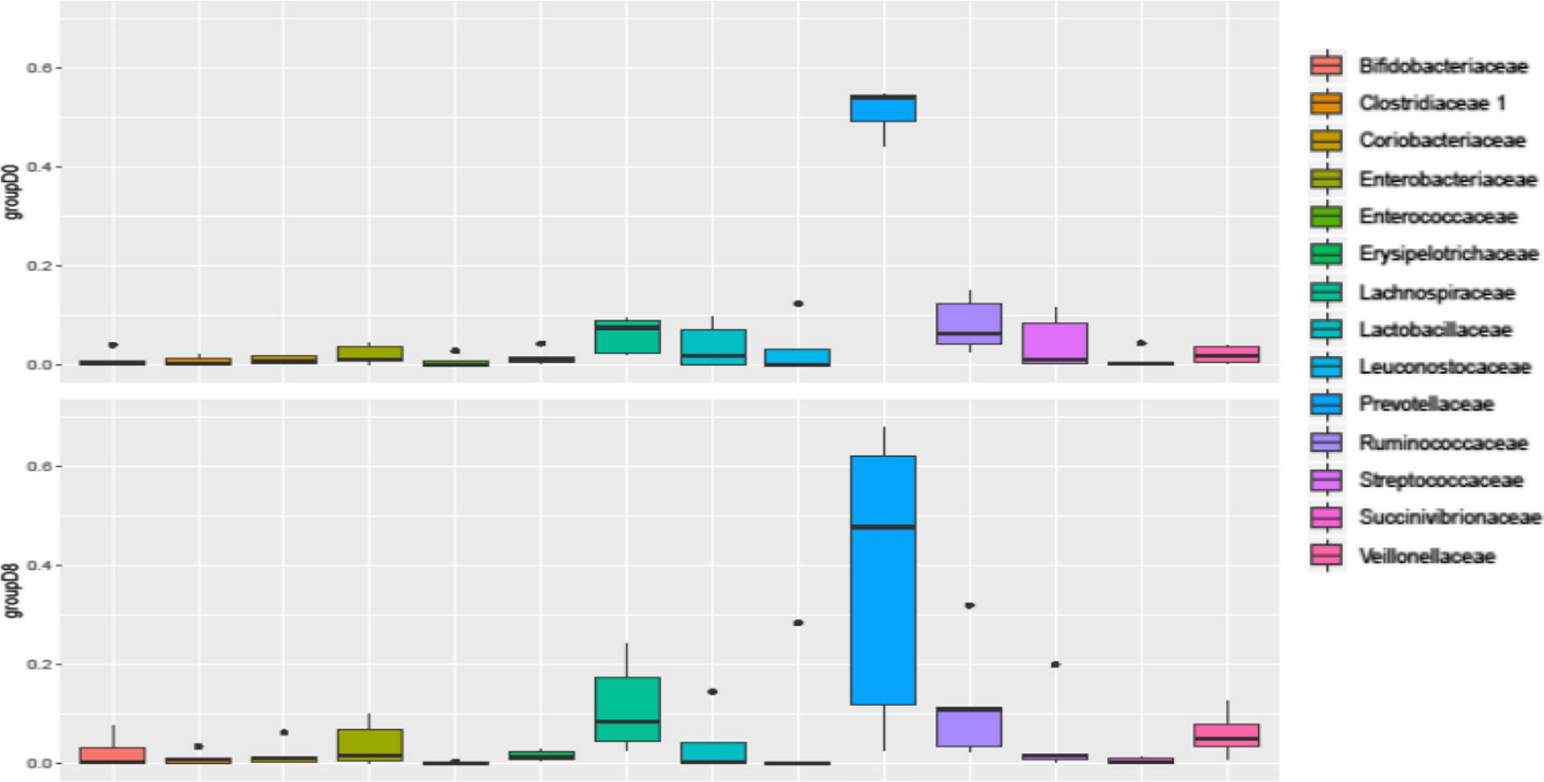
Comparative view of relative abundance of dominant bacterial groups in household children at day 0 and as confirmed for household-contact children of cholera patients at day 8, after the delivery of chlorinated water as part of the cholera-hospital-based water, sanitation, and hygiene (WASH) intervention for 7 days (CHoBI7) at the taxonomic level of family. Relative abundance of bacterial families, e.g., Prevotellaceae, Bifidobacteriaceae, Lachnospiraceae, Clostridiaceae, Enterobacteriaceae and Veilloneleceae increased at day 8, while Lactobacillaceae and Ruminococcaceae remained unchanged

Families of anaerobic bacteria including Prevotellaceae, Bifidobacteriaceae, Lachnospiraceae, Clostridiaceae, and Veilloneleceae in the gut of children after receiving the CHoBI7 WASH program delivery. While these five families were widely distributed, showing high relative abundance at day 8 in all five children, the bacterial families—Lactobacillaceae and Ruminococcaceae remained unchanged; and members of the family Enterobacteriaceae increased in two of the five children receiving CHoBI7 WASH program delivery (Fig. 5).

## Discussions

In countries where safe drinking water is scarce and sanitation is poor, the chlorine-treatment of water was found effective to improve the water quality and reduce child diarrhea [26]. Cholera is a deadly diarrhea that can result in deaths if untreated, and like other diarrhea associated with gut microbiota dysbiosis in young children [21]. The data presented in this study show children receiving the CHoBI7 water treatment and handwashing with soap intervention in cholera patient households had significant reductions in the gut of harmful bacteria belonging to Proteobacteria, and an increase in beneficial Firmicutes. The overall change of the gut microbiota in the reduction of pathogenic bacteria and increase in the beneficial commensals observed in children receiving CHoBI7 intervention was uniform for all five of the children in the present study further demonstrating the public health benefits of the CHoBI7 program, and build on our previous findings that the CHoBI7 intervention significantly reduced cholera [24].

Cholera, caused by *Vibrio cholerae*, is an ancient diarrheal disease that killed millions world-wide. As many as seven cholera pandemics have been recorded since 1817, and is still continuing affecting the world especially in settings where safe drinking water is scarce [32]. For centuries, cholera is endemic in the Ganges delta of Bay of Bengal, Bangladesh and India, where the aquatic habitats are well-established as the niche for *V. cholerae* to thrive and evolve into highly transmissible pathogen undergoing genetics changes in the virulence genes including the cholera toxin (CT), which is the potent toxin responsible for cholera [32]. The successful fecal–oral transmission of an enteric pathogen depends on how successful it is to compete with the host intestinal microbiota, and flourish to cause the infection [33]. The results obtained in the present study showed evidence of the effectiveness of the CHoBI7-chlorine treatment of water in favorably altering the gut microbiota and preventing household transmission of cholera, as there was no cholera among any of the children despite *Vibrionaceae* presence at day 0 reflected *V. cholerae* infection among all five children of the cholera patients’ households.

The chlorine concentration needed to deactivate pathogens in drinking water varies by setting and is based on source-water quality parameters such as turbidity [34]. In a study conducted among the present cohort, we found 0.1 mg/L of free available chlorine needed to deactivate *V. cholerae* and other gram-negative bacteria [26]. Consistent with the efficacy of this level of free chlorine concentration, which was promoted in our intervention, in households of intervention arm, no stored water samples had any detectable *V. cholerae*, and individuals had symptomatic cholera infections [24]. Likewise, our microbiota data showed no presence of bacteria belonging to the family *Vibrionaceae* in the cohort of children drinking chlorine-treated water at day 8. None of the study children developed cholera or any other diarrhea infection during this one-week period, and they were apparently in good health condition. In Dhaka city, most of the low-income groups collect drinking water from various sources, which are unreliable and highly likely to be contaminated with fecal bacteria, including *V. cholerae*, as reported by Rafique et al. [35]. While disinfection of water by boiling is not an easy option for such marginal people, the low-cost point-of-use water treatment by chlorination appears to be an effective method to prevent from cholera, as shown for other waterborne diseases in low-income countries [36–39]. A recent report showed possible contamination of drinking water collected and preserved in the household as it presented with much higher pathogen counts than that found in supply water sources [40]. As per our study, chlorination could ensure post-collection safety of drinking water as the recommended residual chlorine will prevent recontamination of water during collection, transport, and storage at the household [36, 41]. Nonetheless, chlorination of drinking water at the point of collection was shown to significantly reduce childhood diarrhea in low-income groups residing in urban settings with irregular water supply in Bangladesh [42].

In a previous study, we have shown the multitude of multi-drug resistant pathogenic bacteria in the gut of young children in Bangladesh [43]. The predominance of such pathogenic bacteria belonging to phyla Proteobacteria in the gut was associated with poor child growth [44, 45]. Children in our cohort carried six families of bacteria belonging to phylum Proteobacteria, which included *Vibrionaceae* at day 0 in all five children, suggesting household-level transmission of cholera. The observed dysbiosis of microbiota belonging to the six families at day 8, including *Phyllobacteriaceae, Sphingomonadaceae, Erythrobacteraceae, Rhodocyclaceae, Oxalobacteraceae* and *Vibrionaceae* resulted by drinking chlorine-treated water as part of handwashing with soap practices for seven days suggests the potential of CHoBI7 WASH and safe water intervention to prevent cholera among the household members of the disease during the high-risk one-week period. Of 15 Proteobacteria families that were common in all five children before and after drinking chlorinated water, most showed very low relative abundance (< 0.001%), while the predominance of five families including *Enterobacteriaceae*, *Pasteurellaceae, Campylobacteraceae, Helicobacteraceae,* and *Succinivibrionaceae* reflected a high relative abundance in these children. This result appears consistent with the previously published report showing multiple pathogens in the gut of Bangladeshi children [46], attributed presumably to contaminated drinking water and poor living conditions in slums.

Bacteria belonging to the phylum Bacteroidetes are known to possess genes encoding enzymes for carbohydrate metabolism, which is known to help *Bacteroides* to colonize the human gut [47]. Conversely, the phylum Firmicutes includes bacteria that can influence the energy absorption from food; and it has been becoming increasingly evident that the members of the phyla Firmicutes are involved in energy resorption and obesity [48, 49]. Bacteroidetes and Firmicutes are well-known beneficial commensals. Firmicutes to Bacteroidetes ratio (F/B ratio) has been shown to be correlated with obesity and other diseases in human and animal studies [50]. The observed increase of Firmicutes over Bacteroidetes at day 8 was positive in the present study is consistent with our previous study which showed a higher proportion of Firmicutes (43 ± 4; mean ± SEM %) over Bacteroidetes (33 ± 3) with healthy children in Bangladesh (44). A recent microbiota study carried out in Bangladesh was highly successful in improving child health by attracting beneficial gut microbiota via microbiota directed complementary foods (MDCF) [51]. Although the MDCF alone has succeeded in increasing weight for length Z score with positive changes in the level of some plasma proteins in study children, we would propose to combine MDCF study with the CHoBI7 WASH program to improve the gut microbiota composition of young children.

While cholera continues resulting in an estimated 1.3–4.0 million cases, and 21,000–143,000 deaths worldwide [8], the WHO is eying on eliminating the disease from the endemic countries. With that aim, the Global Task Force on Cholera Control (GTFCC) has launched in 2017 a global strategy on cholera control named, Ending Cholera: a global roadmap to 2030, with a target to reduce cholera deaths by 90% [52]. The Ganges Delta of the Bay of Bengal is the historical hotspot of the global cholera where *V. cholerae* shows emergence and succession of lineages demonstrating short-term evolution and success of the bacterium as a natural enemy of people of this region [53]. The National Cholera Control Plan (NCCP) for Bangladesh 2019-2030 is a cholera control strategy, prepared to reach the 90% cholera elimination goal (morbidity and mortality) in the stipulated time (https://www.gtfcc.org/wp-content/uploads/2022/09/national-cholera-plan-bangladesh.pdf). The Ministry of Health and Family Welfare (MOHFW) Bangladesh has taken the following measures to achieve these goal: early case detection and quick response to cholera outbreaks by efficient surveillance, improved case management in all health facilities and controlling endemic situation. A number of development activities particularly oral cholera vaccination (OCV) in hotspots/high-risk areas with water, sanitation & hygiene interventions and surveillance for impact evaluation throughout Bangladesh will contribute to this achievement. This multi-sectoral and multi-year plan will be implemented phase by phase in a period of 12 years with the MOHFW as the lead Ministry and other government sectors and stakeholders supporting and coordinating the implementation. The NCCP for Bangladesh has a demonstration plan as well as a short-, mid-, and long-term objectives. The short-term activities will include sustainable laboratory supported surveillance system, along with early warning and alert response systems (EWARS), improved case management and use of OCV and WASH activities to adopt integrated approach in controlling cholera transmission in the hotspots. In the midterm and the long-term activities, the interventions of the short time activities will be strengthened. As a specific long-term activity, WASH facilities will be gradually expanded nationwide, and our CHoBI7 proved very promising as program in preventing cholera and improving child health in Bangladesh and beyond.

The major limitation of this study was the analysis of fecal samples from a small number of children as this study was nested within the CHoBI7 RCT [24]. Although we have plans to conduct a well-designed follow-up study with a larger cohort in the near future, the data presented in this study on the gut microbiota using next-generation sequencing is the first of its kind to provide a snapshot of the positive changes in gut bacterial community, which we observed in children receiving the CHoBI7 WASH program. Second, our study focused households with an index patient with cholera, and therefore may not be generalizable to other settings. Third, we cannot determine how much of the impact of intervention delivery was attributed to the water treatment versus handwashing with soap component of the intervention since both were combined. This should be explored in future studies.

## Conclusion

The gut microbiota dysbiosis of harmful Proteobacteria and increase of beneficial Firmicutes in household children of an index cholera patient suggest that delivery of a WASH intervention promoting chlorination of drinking water and handwashing with soap can change microbial communities. Future studies are needed that investigate this association in our settings globally.

## Data Availability

The datasets used and/or analyzed during the current study available from the corresponding author on reasonable request.
